# Magnetically Recoverable Ruthenium Catalysts in Organic Synthesis

**DOI:** 10.3390/molecules19044635

**Published:** 2014-04-15

**Authors:** Dong Wang, Didier Astruc

**Affiliations:** ISM, UMR CNRS No. 5255, Université Bordeaux, Talence Cedex 33405, France; E-Mail: wangdong0377@163.com

**Keywords:** magnetic nanoparticles, ruthenium complexes, catalysis, heterogeneous catalysts

## Abstract

Magnetically recyclable catalysts with magnetic nanoparticles (MNPs) are becoming a major trend towards sustainable catalysts. In this area, recyclable supported ruthenium complexes and ruthenium nanoparticles occupy a key place and present great advantages compared to classic catalysts. In this micro-review, attention is focused on the fabrication of MNP-supported ruthenium catalysts and their catalytic applications in various organic syntheses.

## 1. Introduction

In recent years, sustainable and practical chemistry using recyclable catalysts has been one of the most fascinating developments in chemistry in both the academic area and industry [1,2,3,4,5,6,7]. The heterogenization of highly active catalysts on various organic or inorganic supports is probably the most efficient strategy and has gained significant progress towards the achievement of efficient catalyst recovery.

In a related context, the immobilization of catalytic species on MNPs has received considerable attention and is nowadays undergoing an explosive development [8,9,10,11,12]. This is due to the easy preparation of such catalysts and their functionalization, good stability, large surface-to-volume ratio, and efficient recovery procedure by magnetic attraction. The use of MNPs not only offers high catalytic activity and selectivity benefiting from their nanosize, but also fulfills the demands concerning convenient catalyst separation. Recently, MNPs have been successfully used to immobilize a wide variety of transition metal catalysts, organocatalysts, and biocatalysts. These catalysts show sustainable, environmentally benign, and economical characters for various reactions including olefin metathesis, cycloaddition, C-C coupling, hydrogenation, oxidation, reduction, *etc.*

Ruthenium complexes are known in a wide range of oxidation states from −2 to +8 and easily accommodate ligands with various coordination geometries, so that they possess unique opportunities as versatile catalysts [13,14,15,16]. During the past few years, a series of Ru complexes bearing amine, phosphine, oxygen, carbon and hybrid ligands, have been immobilized on MNPs forming magnetically separable catalysts for a variety of reactions, such as asymmetric hydrogenation of aromatic ketones, stereospecific epoxidation, selective oxidation of alcohols and amines, oxidation of levulinic acid to succinic acid, hydration of nitriles, deallylation, asymmetric transfer hydrogenation, redox isomerization of allylic alcohols, heteroannulation of (*Z*)-enynols, olefin metathesis, and synthesis of 1,5-disubstituted 1,2,3-triazoles via azide-alkyne cycloaddition.

In this review, progress in the field of MNP-supported Ru complexes and Ru nanoparticles in organic synthesis is highlighted. At the end of the review, the advantages of magnetically recoverable Ru catalysts, and some of their perspectives for further development are presented.

## 2. MNP-Supported Ru Catalysts for Organic Synthesis

### 2.1. Olefin Metathesis

**Scheme 1:**
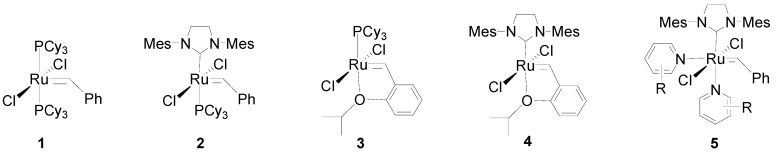
Grubbs-type ruthenium catalysts and derivatives.

Olefin metathesis has been well recognized as a powerful method of generating C=C bonds in modern chemical transformations, especially in the synthesis of polymers, important petrochemicals, and specialty chemicals [17,18,19,20,21,22,23,24,25,26,27,28], since it was discovered by American industrial chemists in the 1960s [29,30,31,32,33]. Olefin metathesis includes ring-opening, ring-closing, and cross metathesis reactions. Ru-based Grubbs-type catalysts **1**–**5** (Scheme 1) [17,18,19,21,23,30,31,32,33,34,35,36,37] are (together with Schrock-type catalysts) widely used in homogeneously catalyzed olefin metathesis, and show superior catalytic activities and extraordinary functional group tolerance. However, the homogeneous Ru catalysts exhibit some inherent drawbacks including the difficult recovery of the catalysts from reaction medium and metal contamination of the products that restrict their possible applications in the pharmaceutical industry and materials science. To overcome these issues, immobilization of the homogenous metathesis catalysts on various supports such as monoliths [38], silica [39,40,41,42], polymers [43,44,45], and MNPs has been proved to be one of the most logical solutions [46]. Among these supports, MNPs have attracted a great interest in olefin metathesis reactions due to their high stability, nano size, and convenient recovery by using an external magnetic field.

**Scheme 2:**
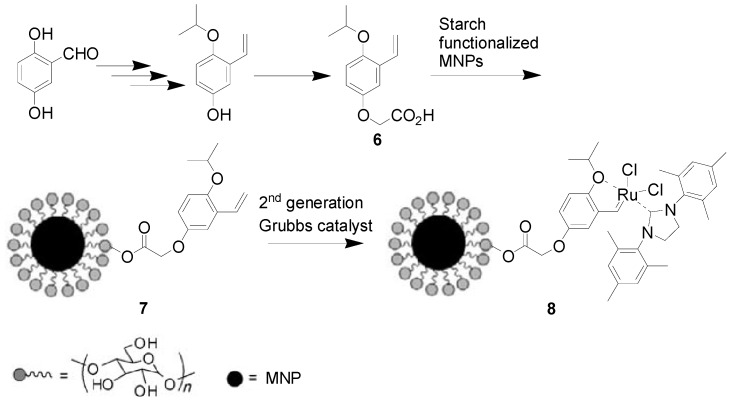
Synthesis of the MNP-supported metathesis ruthenium catalyst **8**.

Zhu *et al.* prepared free and MNP-anchored *ortho*-isopropoxystyrene ligand **6** and **7**, successively, and **2** then reacted with **7** producing MNP-immobilized catalyst **8** (Scheme 2) that possesses a mean diameter size of approximately 100 nm, and Ru content of 0.28 mmol/g. Its catalytic activity was evaluated in both self- and cross-metathesis reactions in terms of yield, TON, TOF [47]. In the case of self-metathesis of fatty acid esters (methyl oleate), **8** provided slightly lower activity than **2** under neat conditions. It was recovered from the reaction mixture by attraction of a magnet with less than 3 ppm Ru leaching. In addition, **8** was recycled for at least five times without any significant decrease in activity. The investigation of cross-metathesis of methyl oleate with methyl acrylate revealed that **8** exhibited much higher and similar activity regarding TOF than the unsupported Grubbs-type ruthenium catalysts. It was magnetically collected and re-used for the next two reaction cycles and maintained the same catalytic performance.

**Scheme 3:**
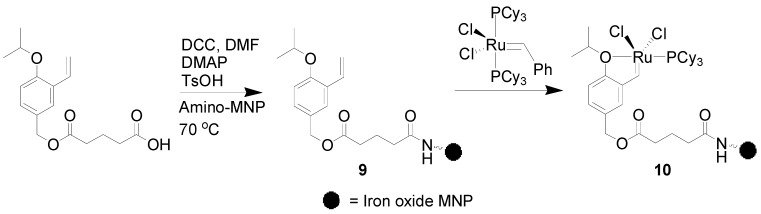
Synthesis of MNP-supported metathesis ruthenium catalyst **10**.

The ruthenium catalyst **10** supported on MNPs was designed and synthesized through the reaction between iron oxide nanoparticles-anchored ligand **9** and Grubbs I catalyst **1** (Scheme 3) [48]. The ring-closing metathesis reactions of a series of substrates were subsequently conducted with **10** (2.5 mol% [Ru]) in CH_2_Cl_2_ at 40 °C. It was found that **10** performed well providing the corresponding cyclic olefins with excellent yields and was recycled up to 22 times without considerable loss in catalytic efficiency.

The Grubbs-III catalyst **5** is highly active for cross metathesis and ring-opening-metathesis polymerization [36,37]. Kirschning’s group [45] demonstrated that **5** was easily immobilized through ligand exchange using polyvinyl pyridine (PVP). The resulting Ru-doped PVP smoothly catalyzed ring-closing metathesis and cross-metathesis reactions at relatively high temperature. The supported catalyst was recyclable in the case of ring-closing metathesis, but an obvious loss of activity was revealed. Emrick *et al.* [49] prepared PEG-functionalized Grubbs III catalyst **5** via ligand exchange, and the catalyst that was obtained was water soluble and effective for ring-opening-metathesis polymerization of norbornene derivatives. Encouraged by the straightforward procedure for immobilization of Grubbs III catalyst, we explored the possibility of anchoring Grubbs III catalyst **5** on MNPs in order to improve the recovery.

**Scheme 4:**
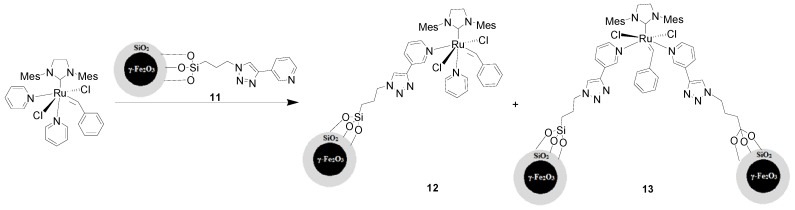
Synthesis of γ-Fe_2_O_3_@SiO_2_ immobilized third generation Grubbs catalysts **12** and **13**.

As shown in Scheme 4, the pre-prepared MNP-supported “click” pyridine ligand **11** (in ten-fold excess) was coordinated to the Ru center of **5** to construct a MNP-enriched Grubbs III catalyst, and the immobilization was confirmed by FT-IR analysis. The supported catalyst should be a mixture resulting from mono- and disubstitution of pyridine ligands by the MNP-derived pyridines in Grubbs-III catalysts (**12**) and (**13**) respectively. The catalytic behavior of this mixture of catalysts was checked for cross metathesis, ring-closing metathesis, and ring-opening metathesis polymerization of olefins. The results showed that only trace of the desired product of cross metathesis reaction between but-3-enenitrile and 1-octadecene was obtained, with 2.5 mol% [Ru] at 40 °C. The ring-closing metathesis reaction of 2,2-diallylmalonic acid diethyl ester did not occur at room temperature in the presence of 2.5 mol% [Ru]. When the temperature was raised to 110 °C, a 34% of yield was obtained, and the catalyst was magnetically recoverable, but deactivated by the third run. In addition, the investigation of ring-opening metathesis polymerization reaction of a norbornene derivative (*cis*-5-norbornene-*exo*-2,3-dicarboxylic anhydride) with the monomer/[Ru] ratio of 13:1, demonstrated that the corresponding polymer was isolated with 80% monomer conversion in 15 h. In conclusion, the catalytic performances of the MNP-supported Grubbs III catalyst for all three metathesis reactions were worse than those of the unsupported Grubbs III catalyst [36,37]. The low catalytic efficiency was attributed to the instability of the coordination between the MNP-immobilized pyridine ligand and Ru, the bulky linker between MNPs and pyridine, and eventually the less efficient substituent group on pyridine concerning metathesis activity [36].

### 2.2. Azide-Alkyne Cycloaddition

The five-membered nitrogen heterocyclic 1,2,3-triazoles have attracted considerable attention in all fields of chemistry, ranging from synthetic organic/inorganic chemistry to pharmaceutical science. Among the numerous methods for 1,2,3-triazole synthesis, azide-alkyne cycloadditions involving Cu [50,51] and Ru [52] catalysis are most efficient ones and they have been widely used for the construction of 1,4- and 1,5-disubstituted 1,2,3-triazoles, respectively.

**Scheme 5:**
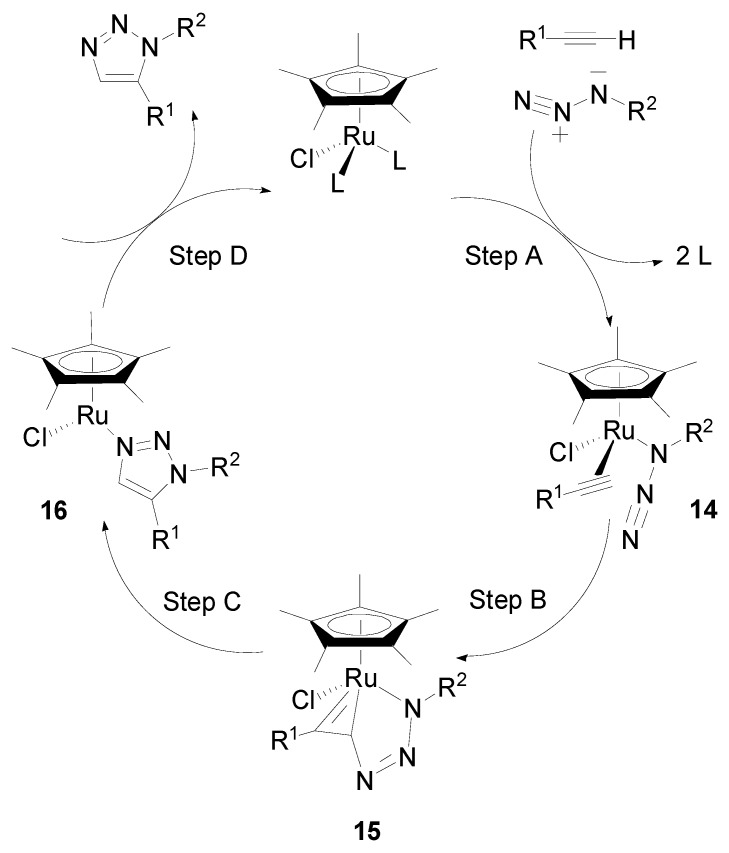
Proposed mechanism for Cp*Ru(II) catalyzed azide-alkyne cycloaddition.

Some Cp*Ru(II) complexes [52,53,54,55] and the cluster (Cp*Ru)_n_ in DMF under microwaves [54] are excellent metal catalysts to regioselectively assemble 1,5-disubstituted 1,2,3-triazoles. As schematically outlined in Scheme 5, the coordination of the starting materials onto the Ru center (step A) produces the Ru intermediate **14** that most certainly undergoes oxidative coupling of the azide and alkyne to give the 6-membered ruthenacycle **15** (step B), which controls the regioselectivity. The next formation of the C-N bond would then occur by reductive elimination yielding the 1,5-disubstituted 1,2,3-triazole, possibly via the coordinated heterocycle **16** (step C). Fokin’s group has reported DFT calculations supporting these mechanistic details [52]. Disubstituted alkynes work as well as terminal alkynes in this RuAAC “click” reaction, whereas only terminal alkynes give the 1,4-disubstituted 1,2,3 triazoles upon Cu-catalysis (CuAAC), because of the required terminal alkyne deprotonation giving a Cu-alkynyl species as an initial step of the latter reaction. The recovery of the Ru catalyst, however, remains a long-standing problem. Viewing economy and environmental benefit, it is essential to develop investigations of the suppression of heterogeneous Ru contamination by Ru(II) complexes upon Ru separation following the synthesis of 1,2,3-triazoles.

**Scheme 6:**
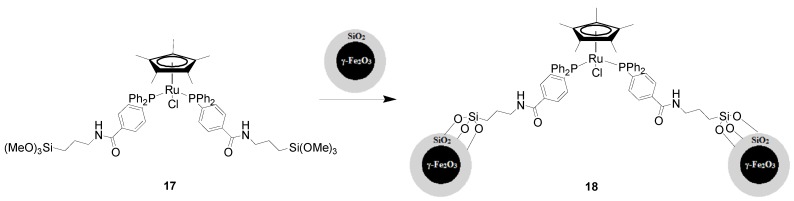
Synthesis of Cp*(PPh_3_)_2_Ru/SiO_2_/γ-Fe_2_O_3_ **18**.

**Scheme 7:**
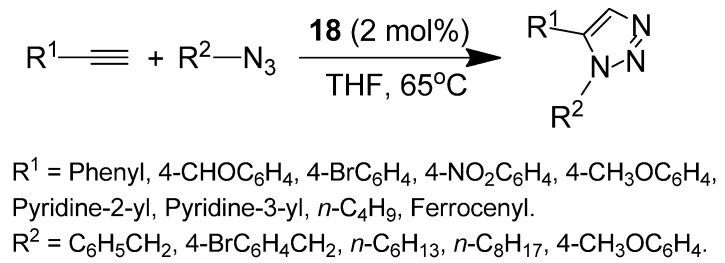
RuAAC reactions in the presence of the magnetic catalyst Cp*(PPh_3_)_2_Ru/SiO_2_/γ-Fe_2_O_3_ **18**.

Our group has reported the first example of MNP-supported Cp*(PPh_3_)_2_Ru(II) catalyst for azide-alkyne cycloaddition (AAC) [56]. The Si(OMe)_3_-functionalized Cp*(PPh_3_)_2_Ru complex **17** was obtained via coordination of Si(OMe)_3_-functionalized PPh_3_ with the (Cp*RuCl_2_)_n_ cluster. Subsequently, core-shell γ-Fe_2_O_3_@SiO_2_ nanoparticles with an average size of 30 nm were successfully enriched with **17** by coupling reaction as shown in Scheme 6. This catalyst **18** was initially evaluated in AAC using phenylacetylene and benzyl azide as model substrates with 2 mol% [Ru] in THF. The corresponding 1,5-disubstituted 1,2,3-triazole was synthesized in 91% yield and over 99.9% selectivity within 3 h. Then, the catalyst **18** was easily removed from the reaction medium by magnetic attraction and recycled at least five times with a gradual slight loss of activity (down to 77%), and a slight decrease in selectivity for the 1,5-disubstituted 1,2,3-triazole product. The substrate scope was then investigated using aryl, aliphatic, and ferrocenyl acetylenes that exhibited good reactivities with benzyl azide in the presence of **18**. The aliphatic azides and benzyl azides bearing a Br substituent are also suitable cycloaddition partners; when aryl azide (*p*-methoxyphenyl azide) was employed, the yield of 1,5-disubstituted 1,2,3-triazole was somewhat lower (Scheme 7). The catalyst **18** was also active with internal alkynes such as 1,2-diphenylethyne, and the 1,4,5-trisubstituted 1,2,3-triazole product was obtained in 77% yield.

### 2.3. Hydrogenation

Hydrogenation reactions, in particular asymmetric hydrogenations, have been widely studied, because they are among the most versatile reactions in all fields of chemistry from pharmaceutical science to petrochemistry, Recently, MNP-immobilized Ru complexes were shown to be efficient catalysts for asymmetric or symmetric hydrogenation of unsaturated compounds.

**Scheme 8:**
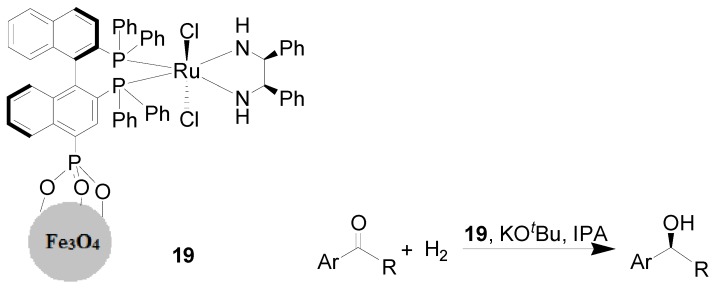
Asymmetric hydrogenation of aromatic ketones in the presence of the MNP-supported chiral Ru catalyst **19**.

Fe_3_O_4_ nanoparticles were readily prepared by the coprecipitation method, and these MNPs were then successfully used for the immobilization of the as-synthesized Ru(BINAP-PO_3_H_2_)(DPEN)Cl_2_ complex, forming MNP-anchored chiral Ru catalyst **19**. This heterogeneous catalyst afforded high catalytic activity and enantioselectivity in the asymmetric hydrogenation of aromatic ketones in the presence of KO*^t^*Bu under 700 psi of hydrogen pressure (Scheme 8) [57]. A series of secondary alcohols were generated through hydrogenation of their corresponding aromatic ketones over 0.1 mol% of **19**, with 100% conversion and remarkably high *e.e.* values compared with its homogeneous counterpart Ru(BINAP-PO_3_H_2_)(DPEN)Cl_2_. Furthermore, after completion of the reactions, the heterogeneous catalyst was magnetically recovered and reused for 14 times without noticeable loss in both conversion and *e.e.* value. In this report, the synthesis of other Fe_3_O_4_ nanoparticles was also reported to involve the use of thermal decomposition, and the supported Ru complex showed lower durability (being only reused for four cycle runs) in comparison with the above-mentioned catalyst **19**.

**Scheme 9:**
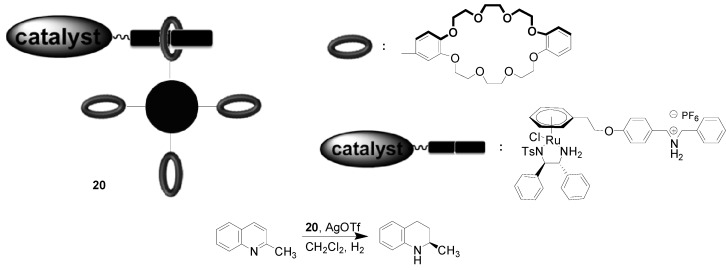
Asymmetric hydrogenation of 2-methylquinoline in the presence of **20**.

Fan’s group [58] reported a novel magnetically separable Ru catalyst **20** containing a host-guest assembly, in which dibenzo[24]crown-8-modified Fe_3_O_4_ nanoparticles was used as a host, and a dialkylammonium salt tag connected with (η^6^-arene)[*N-*(*para*-toluenesulfonyl)-1,2-diphenyl- ethylenediamine]ruthenium trifluoromethanesulfonate [Ru(OTf)(TsDPEN)(η^6^-arene)] was regarded as a guest (Scheme 9). The catalytic performance of **20** was evaluated in the asymmetric hydrogenation of 2-methylquinoline, and the reaction was carried out using 2 mol% of **20** in the presence of AgOTf under 50 atm H_2_ at 150 °C in CH_2_Cl_2_. The corresponding hydrogenated compound was produced with full conversion and 89% of *e.e.* value. On the basis of the formation of a pseudorotaxane complex between the host and the guest, the Ru catalyst was easily collected from the reaction medium by using an external magnetic decantation, and reused for at least 5 runs without significant decrease in activity and enantioselectivity.

**Scheme 10:**
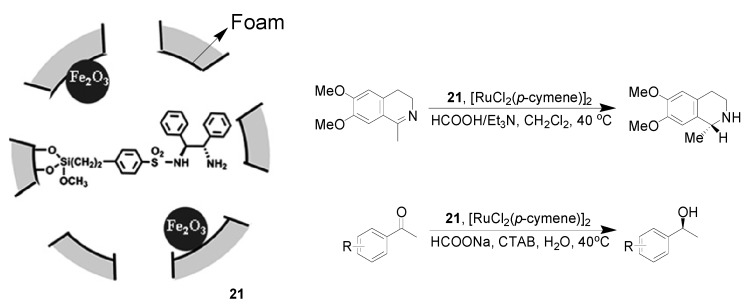
Asymmetric hydrogenation of aromatic ketones using catalyst Ru-**21**.

Transfer hydrogenation is considered to be one of the most important branches of hydrogenation, and it has received more and more attention, because of the easy availability of reductants, its high performance, operational simplicity, and low cost [59,60]. In this field, Ru-TsDPEN (TsDPEN = *N*-(*p*-toluenesulfonyl)-1,2-diphenylethylenediamine) is perhaps the most popular chiral catalyst, with the use of 2-propanol, HCOOH–Et_3_N mixture and aqueous HCOONa as hydrogen donors. Recently, a magnetic siliceous mesocellular foam material-encapsulated Ru-TsDPEN derived catalyst was developed. Starting from siliceous mesocellular foam, the functionalization with γ-Fe_2_O_3_ and TsDPEN provided the magnetic siliceous mesocellular foam-caged TsDPEN ligand **21** (Scheme 10). The catalytic property of **21-**[RuCl_2_(*p*-cymene)]_2_ was initially tested in the asymmetric hydrogenation of substituted dihydroisoquinoline using HCOOH–Et_3_N azeotrope (molar ratio 2.5/1, pH 3.1) as hydrogen donor. The reaction gave 98% yield and 94% *e.e.* values, which were comparable with the result of the use of homogeneous Ru-TsDPEN. The catalyst was then successfully extended to the asymmetric hydrogenation of aromatic ketones with HCOONa-H_2_O as hydrogen donor. Various secondary alcohols were produced with 99% conversions and 89%–97% *e.e.* values. Moreover, this combination of **21** and [RuCl_2_(*p*-cymene)]_2_ allowed the Ru catalyst to be simply recovered with an external magnet and reused consecutively for at least nine runs, while maintaining nearly the same activity and enantioselectivity [61].

**Scheme 11:**
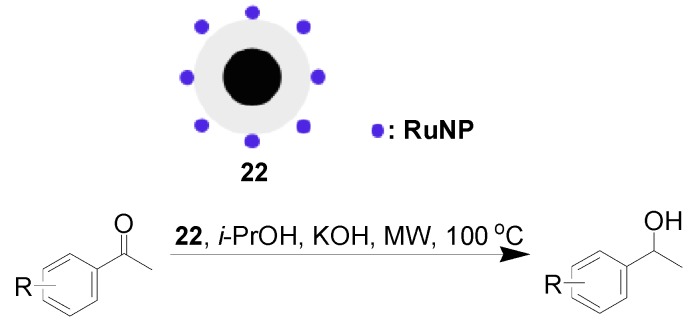
Magnetic silica-supported RuNPs: An efficient catalyst for transfer hydrogenation of carbonyl compounds.

Varma *et al.* [62,63] demonstrated that MNP-supported RuNPs were competitive candidates for the catalysis of transfer hydrogenation of carbonyl compounds. RuNPs supported on NiFe_2_O_4_ were readily prepared and utilized for transfer hydrogenation of a range of carbonyl compounds with isopropyl alcohol as hydrogen donor under microwave irradiation conditions. The desired hydrogenated compounds were isolated in 90%–98% yields. The supported catalyst showed good recyclability. After magnetic collection, it was recycled for another four runs, and its activity remained high [62]. In another report, the same group achieved the assembly of RuNPs on Fe_3_O_4_@SiO_2_ nanoparticles from Fe^2+^, Fe^3+^ and Ru^3+^ precursors in one-pot. The transfer hydrogenation of acetophenone was conducted using isopropanol as a solvent and KOH as base using the obtained hybrid nanocatalyst **22** as catalyst under microwaves irradiation. Within 30 min, acetophenone was quantitatively converted to the corresponding alcohol. A wide range of secondary alcohols were synthesized in good to excellent yields under the same conditions (Scheme 11) [63]. In the case of the transfer hydrogenation of acetophenone, after the completion of the first reaction, catalyst **22** was collected magnetically and successfully recycled for at least 3 times with the same yield. ICP-AES and TEM analyses revealed that no Ru metal was detected in the reaction medium after completion of the reaction, and the catalyst nearly remained with the same size and morphology during the first three reaction cycles.

### 2.4. Oxidation

Oxidation is of paramount importance in both academic and industrial synthetic chemistry. MNP-immobilized Ru catalysts have received considerable attention for the oxidation of alcohols, amines, levulinic acid, and special alkylarene (xanthenes).

**Scheme 12:**
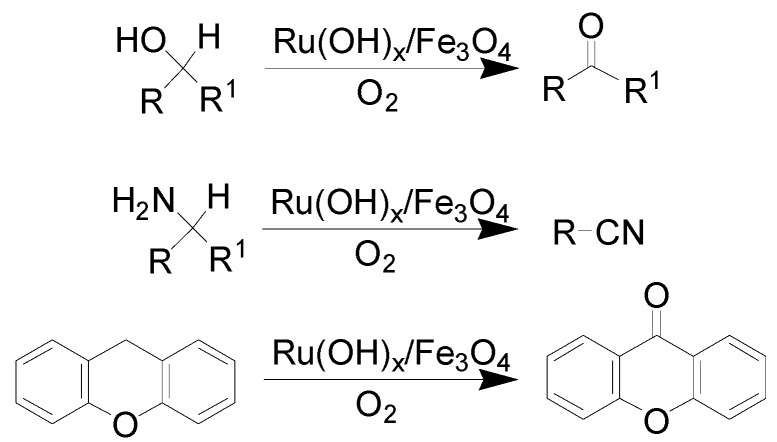
Oxidation of alcohols and amines over Ru(OH)_x_/Fe_3_O_4_ nanoparticles.

Ruthenium hydroxide supported on Fe_3_O_4_ nanoparticles (Ru(OH)_x_/Fe_3_O_4_) was easily prepared and exhibited high catalytic performances in aerobic oxidation of alcohols, amines, and xanthene (Scheme 12) [64,65]. The oxidation of various alcohols was efficiently conducted with 3.8 mol% of [Ru] under 1 atm of molecular oxygen, and the corresponding aldehydes and ketones were provided in excellent yields and almost 100% selectivity [64]. The catalytic system was then successfully extended to the oxidation of amines to form nitriles, and high yields were generally detected. However, small amounts of *N*-alkylimines were also observed as byproducts in the process. In addition, xanthene was also quantitatively oxidized to 9-xanthenone with >99% yield under the same conditions [64]. The recyclability test revealed that almost all the Ru(OH)_x_/Fe_3_O_4_ catalyst was removed from the reaction medium in each case and continuously used for other reaction cycles.

**Scheme 13:**
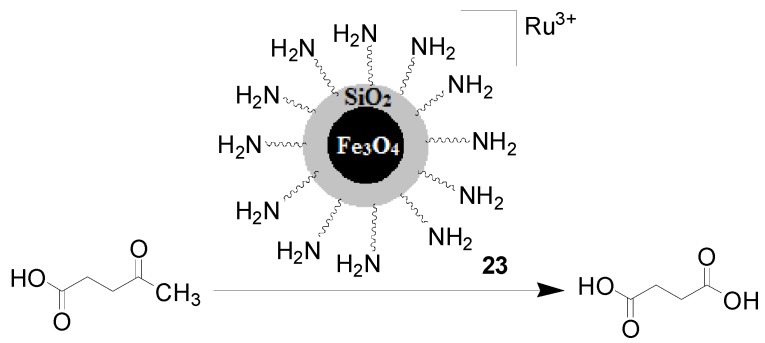
Ru-based magnetic nanoparticles (MNP) for succinic acid synthesis from levulinic acid.

Coman and coworkers [66] reported that the oxidation of levulinic acid to succinic acid was efficiently promoted by Ru(III)/functionalized silica-coated magnetic nanoparticles **23** (Scheme 13 ). This catalyst was easily prepared through three-step synthesis including silica protection of Fe_3_O_4_ nanoparticles, functionalization with aminopropyl groups, and coordination with RuCl_3_. The catalytic performance of **23** strongly depends on the pressure of oxygen, reaction temperature and solvent. The reaction reached 53.8% conversion and 96% selectivity towards succinic acid under 10 bar of oxygen at 150 °C in water within 6 h (Scheme 13). The use of lower pressure of oxygen, lower reaction temperature and other solvents decreased the conversion of levulinic acid, however. Furthermore, this heterogeneous catalyst was consecutively reused at least four times, with conversion ranging from 53.5% to 58%, and selectivity ranging from 93.4% to 98.5%. ICP analysis showed that only negligible amounts of Ru leached from the initial catalyst, which indicated the high stability of the catalyst **23**. The authors mentioned that the actual catalytic species for the oxidation is perhaps [Ru(H_2_O)_5_OH]^2+^ that was generated by the reaction of Ru species with H_2_O, but no evidence was offered to confirm this proposition.

A Ru(III)/amine-functionalized Fe_3_O_4_@SiO_2_ nanocatalyst with a mean diameter of 60 nm was evaluated in the oxidation of alcohols, and it was shown that a series of carbonyl compounds were obtained with excellent conversions and over 99% selectivity, in the presence of 3 atm oxygen at 100 °C with 4 mol% [Ru]. Interestingly, the magnetic Ru(0) NPs that were generated by reduction of the present magnetic Ru(III) catalyst were able to catalyze the hydrogenation of cyclohexene giving full conversion and TOF of 420 h^−1^, under 6 atm hydrogen at 75 °C [67]. In both cases of oxidation and hydrogenation, the amounts of leaching Ru were negligible, which was attributed to the powerful coordination ability of amino group to Ru. This report strongly demonstrates the versatility of Ru catalysts.

### 2.5. Nitrile Hydration

Functionalized amides are key intermediates that are frequently used in various chemical fields, and nitrile hydration is the one of the most important technologies for the large-scale synthesis of amides. In order to achieve this transformation, MNPs-anchored Ru complexes were recently designed and applied as catalysts.

**Scheme 14:**
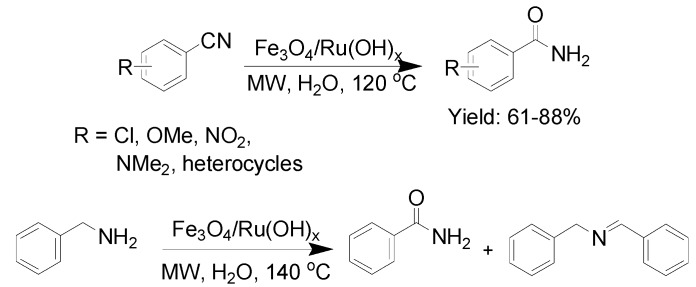
Hydration of nitrile using Fe_3_O_4_/Ru(OH)_x_.

Amine-modified MNPs were synthesized through sonicating Fe_3_O_4_ nanoparticles with dopamine. The obtained functionalized MNPs then coordinated RuCl_3_ at a basic pH, constructing the hybrid nanoparticles decorated with Ru(OH)_x_. This nanomaterial was explored as a catalyst for the hydration of nitriles in aqueous medium under microwave irradiation [68]. In the initial experiment, hydration of benzonitrile was chosen as a model reaction, and the desired amide was produced in 85% yield after 30 min of microwave irradiation at 130 °C in water. Furthermore, with this model reaction, the magnetic Ru catalyst was efficiently recovered by using a handheld magnet and reused for at least 3 reaction cycles without obvious loss of activity. Using the same catalytic system, 14 amides were synthesized in 61%–88% yields (Scheme 14). The scope of this strategy was also tested for the oxidation of benzyl amine, and a mixture of corresponding amide and benzylidenebenzylamine were generated (Scheme 14). The percentage of benzylidenebenzylamine in the mixture increased up to 78% upon prolonging the time of microwave irradiation. A subsequent report from the same group [69] demonstrated that Ru(OH)_x_ supported on Fe_3_O_4_ nanoparticles was readily prepared from Fe^2+^, Fe^3+^, and Ru^3+^ precursors in one-pot, and showed a highly efficient activity and selectivity in the hydration of nitrile.

**Scheme 15:**
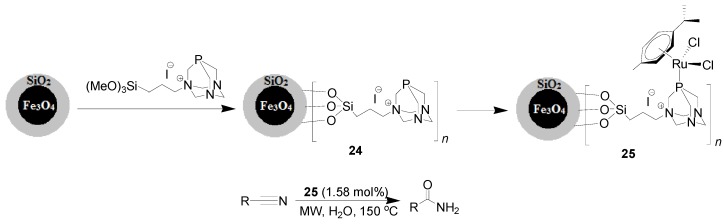
Hydration of nitriles catalyzed by the Ru complex-functionalized MNP **25**.

Ruthenium(II)-arene derivatives bearing the phosphane 1,3,5-triaza-7-phosphatricyclo[3.3.1.1] decane (abbreviated as RAPTA) is a classical organometallic compound with versatile applications in catalysis [70,71,72]. The groups of Basset and Polshettiwar [73] firstly reported the immobilization of RAPTA on MNPs. The synthetic procedure involves the preparation of MNPs-anchored PTA ligand **24** upon reaction of SiO_2_-coated Fe_3_O_4_ with trimethoxysilane-functionalized PTA ligand. Further reaction of **24** with a slight excess of the commercially available Ru precursor [RuCl(μ-Cl)(η^6^-*p*-cymene)_2_] provided the magnetic Fe_3_O_4_–RAPTA nanoparticles **25** that was subsequently evaluated in the hydration of nitriles (Scheme 15). Under microwaves irradiation, 55 amides bearing a broad scope of substituting groups were efficiently isolated using 1.58 mol% of [Ru] within short time, with excellent GC yields. Aiming to seek the possibility of practical application, the recyclability of **25** was examined based on the hydration of both benzonitrile and 2-phenoxyacetonitrile. The catalyst **25** was simply separated using an external magnetic field, and continuously used for 4 and 5 times with slight decrease in yield.

All the above-mentioned processes of hydration of nitriles to amides over MNPs-supported Ru catalysts are truly green and sustainable due to the use of environmentally friendly water as the reaction medium, the use of alternative microwave energy source, and their excellent recyclability.

### 2.6. Other Reactions

**Scheme 16:**
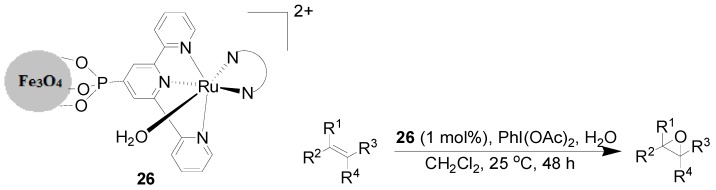
Epoxidation of selected alkenes catalyzed by molecular ruthenium complexes anchored on MNPs.

Other organic transformations catalyzed by MNP-immobilized Ru catalysts include redox isomerization of allylic alcohols, heteroannulation of (*Z*)-enynols, deallylation, trimethylsilylation of alcohols and phenols, and hydrolysis reactions. The highly active and selective homogeneous epoxidation catalyst [Ru(trpy-P)(B)(H_2_O)]^2+^ (trpy-P is diethyl [2,2':6',2'-terpyridin]-4'-ylphosphonate, B = bpm) was immobilized on Fe_3_O_4_ nanoparticles. The resultant heterogeneous catalyst **26** displayed practically the same behavior as its homogeneous counterpart in the epoxidation of alkenes (Scheme 16) [74]. A series of epoxides were synthesized in moderate to good both yields and selectivity towards *cis*-epoxides. The catalyst **26** exhibited an outstanding recyclability and could keep with a similar catalytic performance in terms of activity and selectivity for more than 5 runs.

**Scheme 17:**
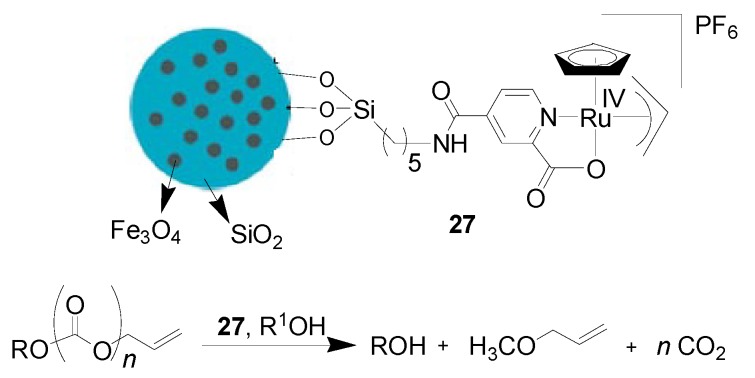
Cleavage of allyl esters catalyzed by the magnetically recoverable complex [CpRu(η^3^-C_3_H_5_)(2-pyridinecarboxylato)]PF_6_.

The preparation and catalytic application of the complex [CpRu(η^3^-C_3_H_5_)(2-pyridinecarboxylato)]PF_6_ supported on micro-size spherical Fe_3_O_4_@SiO_2_ particles were reported by Kitamura’s group [75]. The as-synthesized magnetic catalyst **27** with good dispersibility powerfully promoted the cleavage of allyl esters in alcoholic solvents in the absence of any extra additives (Scheme 17). Multiple recycling experiments for the cleavage of allyl esters involving magnetic decantation of the catalyst were carried out using **27** with slight loss of activity. Below 0.2% of Ru leaching was detected in each reaction cycle. The results presented here should further enhance the utility of this heterogeneous catalyst in protecting group chemistry.

**Scheme 18:**
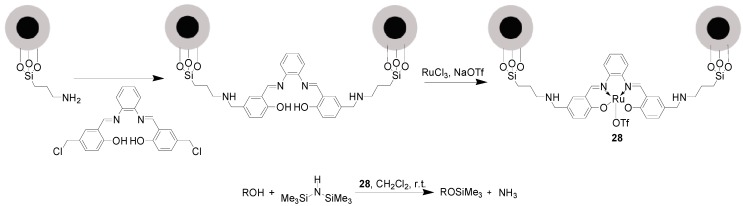
Trimethylsilylation of alcohols and phenols with HMDS catalyzed by **28**.

Starting from NH_2_-modified MNPs, the MNPs-supported complex [Ru^III^(Salophen)OTf], **28**, was assembled via the successive reactions of NH_2_-modified MNPs with H_2_Salophen, RuCl_3_ and NaOTf (Scheme 18) [76]. This catalyst **28** exhibited remarkable catalytic performances for the trimethylsilylation of primary and secondary alcohols as well as phenols with hexamethyldisilazane (HMDS). Benzylic alcohols bearing both electron-donating and electron-withdrawing groups smoothly reacted with HMDS over 4 mol% of [Ru], producing the corresponding TMS ethers in 96%–100% yields in a short time at room temperature. Linear, secondary, tertiary alcohols, and phenols were also suitable participants for the trimethylsilylation, the desired TMS ethers being provided in high yields. However, the reaction times were longer in comparison with those involving benzylic alcohols. Importantly, catalyst separation was easily achieved using an external magnet without any Ru leaching, and the recovered catalyst was recycled for at least 5 runs without loss in catalytic performance.

Bimetallic transition metal core–shell nanoparticles (NPs) have a bright future in catalysis due to their enhanced stability, activity, and other properties compared to their monometallic counterparts [77,78]. Ma *et al.* [79] pioneered the synthesis of bifunctional catalytic and magnetic Ni@Ru core–shell NPs through the seeded-growth method; meanwhile monometallic NiNPs, RuNPs were prepared. All the as-synthesized NPs as well as a physical mixture of NiNPs and RuNPs were used as catalysts in the hydrolysis of ammonia–borane (AB). NiNPs were found to be inactive for this transformation; both monometallic Ru and the physical mixture were active, but with similar level of activity. Interestingly, Ni@Ru NPs with the same amount of [Ru] as in the monometallic Ru and in the physical mixture exhibited remarkably enhanced catalytic performances, which was attributed to the much smaller size of RuNPs in Ni@Ru NPs than in monometallic RuNPs (2.5 nm *vs.* 8 nm), the increased stability of the deposited RuNPs, and the interaction between NiNPs and RuNPs on the electronic structure of the active metal in Ni@Ru NPs. Importantly, the recyclability test revealed that the Ni@Ru NPs catalyst was able to be magnetically collected and used for 3 more cycles with slight decrease in activity.

## 3. Conclusions and Perspectives

MNPs represent a bridge between homogeneous and heterogeneous catalysis, and are a family of prospective materials with a bright future. To date, MNP-supported ruthenium catalysts have been readily prepared and efficiently used as catalysts in olefin metathesis, azide-alkyne cycloaddition, hydrogenation, oxidation, nitrile hydration, and several other reactions. The use of MNPs shows many advantages such as convenient separation, efficient recovery, and similar or higher activity compared to their homogeneous counterparts. These strategies for immobilizing Ru complexes on MNPs open a broad field of application of Ru complexes toward “green” chemistry.

Although remarkable progress has been made, only selected Ru complexes were involved in these reactions until now, and not all catalysts have provided satisfactory results. We believe that a fast-increasing number of multi-functionalized MNPs and useful methods will be probed, developed and used for the immobilization of various Ru complexes in various catalytic reactions. Further work is also required to extend these sustainable catalysts towards use in industrial production.
